# Supplementary material for: Splice-Junction-Based Mapping of Alternative Isoforms in the Human Proteome
Source: Cell Rep. Author manuscript; Available in PMC 2020 Jan 15. (PMC6961840; doi:10.1016/j.celrep.2019.11.026)
Supplement: 3 [file NIHMS1546469-supplement-3.zip › DF2/PXD0010154/Spleen-56-O60229-IFDNDPTQDEMSLEGSSYR.pdf]

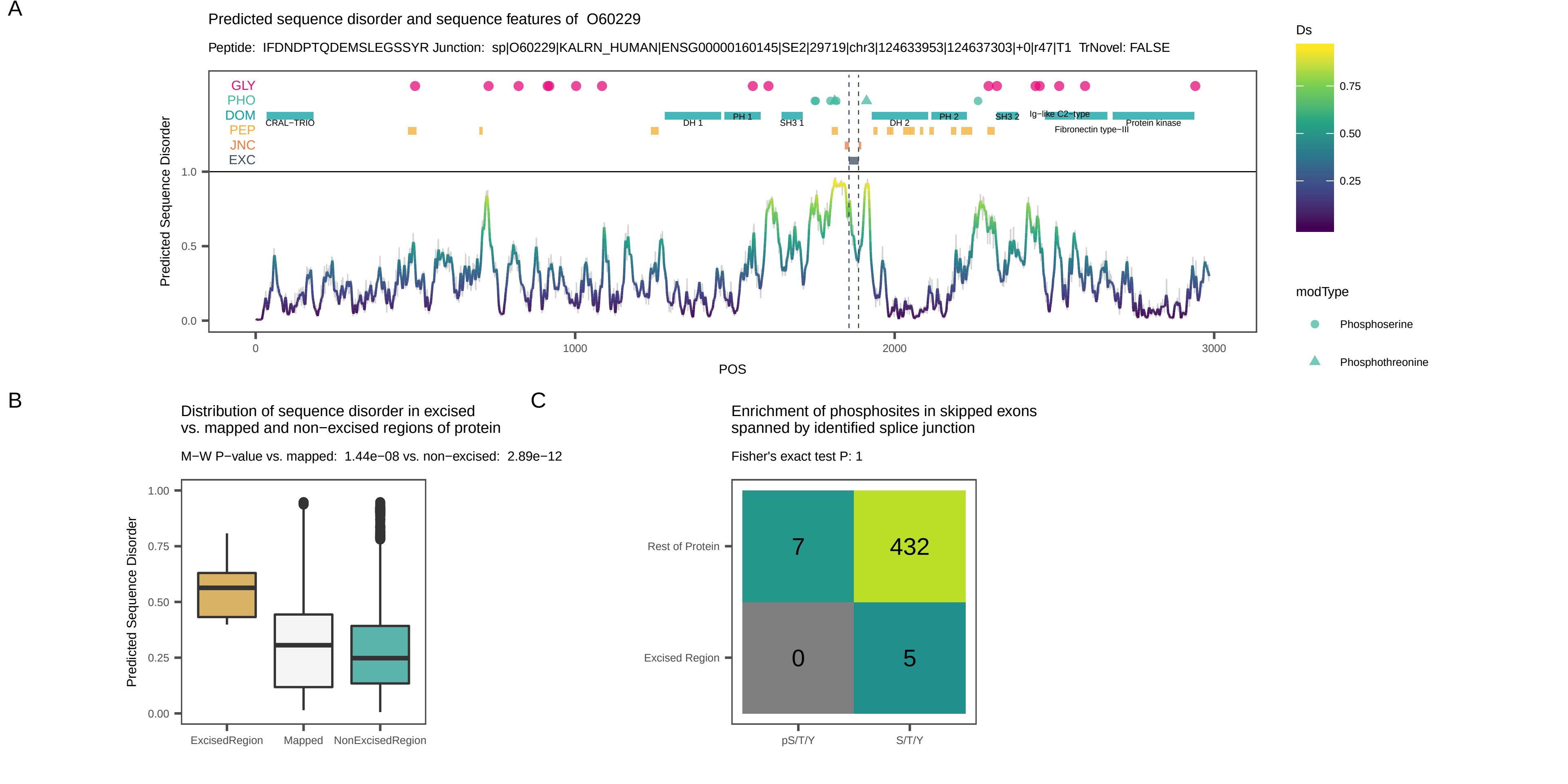
A
Predicted sequence disorder and sequence features of O60229
Peptide: IFDNDPTQDEMSLEGSSYR Junction: sp|O60229|KALRN_HUMAN|ENSG00000160145|SE2|29719|chr3|124633953|124637303|+0|r47|T1 TrNovel: FALSE
GLY
PHO
DOM
PEP
JNC
EXC
CRAL−TRIO
DH 1
PH 1
SH3 1
DH 2
PH 2
SH3 2
Ig−like C2−type
Fibronectin type−III
Protein kinase
Predicted Sequence Disorder
1.0
0.5
0.0
0
1000
2000
3000
POS
Ds
0.75
0.50
0.25
modType
Phosphoserine
Phosphothreonine
B
Distribution of sequence disorder in excised vs. mapped and non−excised regions of protein
M−W P−value vs. mapped: 1.44e−08 vs. non−excised: 2.89e−12
Predicted Sequence Disorder
1.00
0.75
0.50
0.25
0.00
ExcisedRegion
Mapped
NonExcisedRegion
C
Enrichment of phosphosites in skipped exons spanned by identified splice junction
Fisher's exact test P: 1
Rest of Protein
Excised Region
7
432
0
5
pS/T/Y
S/T/Y
